# Quality assessment metrics for whole genome gene expression profiling of paraffin embedded samples

**DOI:** 10.1186/1756-0500-6-33

**Published:** 2013-01-30

**Authors:** Douglas W Mahoney, Terry M Therneau, S Keith Anderson, Jin Jen, Jean-Pierre A Kocher, Monica M Reinholz, Edith A Perez, Jeanette E Eckel-Passow

**Affiliations:** 1 Biomedical Statistics and Informatics, Mayo Clinic, 200 First Street SW, Rochester, MN 55905, USA; 2 Clinical Studies & Translational Diagnostics, Ventana Medical Systems, Inc., 1910 E. innovation Park Drive, Tucson, AZ, 85755, USA; 3 Hematology Oncology, Mayo Clinic, 4500 San Pablo Road, Jacksonville, FL, 32224, USA

**Keywords:** High-dimensional array quality, Formalin-Fixed, Paraffin-embedded tissue, Outlier detection

## Abstract

**Background:**

Formalin fixed, paraffin embedded tissues are most commonly used for routine pathology analysis and for long term tissue preservation in the clinical setting. Many institutions have large archives of Formalin fixed, paraffin embedded tissues that provide a unique opportunity for understanding genomic signatures of disease. However, genome-wide expression profiling of Formalin fixed, paraffin embedded samples have been challenging due to RNA degradation. Because of the significant heterogeneity in tissue quality, normalization and analysis of these data presents particular challenges. The distribution of intensity values from archival tissues are inherently noisy and skewed due to differential sample degradation raising two primary concerns; whether a highly skewed array will unduly influence initial normalization of the data and whether outlier arrays can be reliably identified.

**Findings:**

Two simple extensions of common regression diagnostic measures are introduced that measure the stress an array undergoes during normalization and how much a given array deviates from the remaining arrays post-normalization. These metrics are applied to a study involving 1618 formalin-fixed, paraffin-embedded HER2-positive breast cancer samples from the N9831 adjuvant trial processed with Illumina’s cDNA-mediated Annealing Selection extension and Ligation assay.

**Conclusion:**

Proper assessment of array quality within a research study is crucial for controlling unwanted variability in the data. The metrics proposed in this paper have direct biological interpretations and can be used to identify arrays that should either be removed from analysis all together or down-weighted to reduce their influence in downstream analyses.

## Background

Many institutions have large archives of formalin-fixed paraffin-embedded (FFPE) tissue. Compared to the general availability, sample collection protocols, and time-sensitive nature of fresh-frozen tissue, these large archives of FFPE tissues are easily assessable and provide a unique opportunity for understanding genomic signatures of disease on a large scale as well as the ability to evaluate long-term prognostic associations [1,2]. These FFPE samples have been relatively untouched by high dimensional platforms due to RNA degradation and cross-linking of nucleic acids due to formalin fixation process [3]. However, Illumina introduced their cDNA-mediated Annealing Selection extension and Ligation (DASL) assay that is specifically designed to enable whole genome expression profiling using degradated RNA and is used in conjunction with their BeadArray technology [4-7]. Similarly, the Ovation® FFPE WTA system is available from NuGEN for processing archival tissues to be analyzed by the Affymetrix platform. Although sequencing-based technologies are seen by many as a better alternative to microarray-based methods, sequencing is limited by difficult sample preparation protocols for FFPE samples and the cost of large-scale studies. In addition, several works have reported on the validity of microarray-based approaches to FFPE relative to fresh-frozen tissue and the growth for this technology will most likely increase rapidly [8-10].

**Figure 1:**
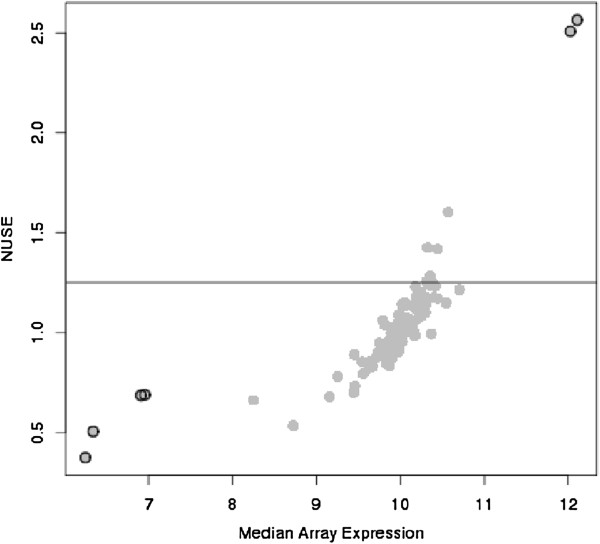
**Plot of *NUSE *vs. median array expression.** The circled points represent arrays that were Figure 1: Plot of *NUSE* vs. median array expression. The circled points represent arrays that were considered to be of poor quality by *Stress/dfArray* and the horizontal line represents the cutoff suggested for *NUSE*. The bead level standard deviations information was available from one plate of the experiment (n=96).

The prevailing consensus in the literature is that normalization is a necessary step for microarray platforms in order to adjust for the influence of factors extraneous to the primary biological question such as sample preparation, scanner efficiency, and cross hybridization of probes [11,12]. A fundamental premise of many of the normalization routines is an assertion that the true overall distribution of RNA abundance will be essentially identical from sample to sample, since only a minority of genes will be differentially expressed. Quantile normalization, for instance, forces the marginal distributions across arrays to be equivalent, while other routines use feature specific estimates as a normalization target in order to estimate non-linear bias correction functions [13,14]. Typically, the distribution of pre-normalized data is well behaved and the normalization corrections are numerically small. For array data using FFPE samples, our experience is that the distributional properties of pre-normalized intensity values are extremely variable in the amount of abundance, skewness, and spread that is present in the data (Figure 1). A primary challenge for proper analysis of these data is defining a reasonable target distribution to normalize against without adding unwanted variation to the biological signals by including outlier arrays in the normalization process.

Although normalization will equalize the distribution of feature intensities across the arrays, there remains a need to assess the quality of the data. For example, of 7 FFPE experiments submitted to Gene Expression Ominbus (GSE20140, GSE19977, GSE23368, GSE20017, GSE25727, GSE28064, and GSE21921) only the latter two studies acknowledged that array quality assessments were even conducted and neither of these two studies reported their findings [1,2,8,9,15-17]. Recently Chow et al. reported on their workflow of assessing array quality for FFPE samples using the *lumi* pipeline [18]. Although this work is an important initial step towards assessing the quality of array data using FFPE samples, the metrics used are based on measures of multidimensional dissimilarity; a concept that may be unfamiliar to the average researcher. Furthermore, thresholds for declaring a sample to be an outlier is study specific and thus make inter-study interrogation difficult.

In this work, we introduce two metrics that easily can be used to assess microarray quality regardless of the platform under consideration and have direct clinical interpretations. These two metrics are used 1) to measure how much data from a single microarray needs to be “stretched” during the normalization process in order to make its marginal distribution match with the remaining arrays (*Stress*) and 2) a measure of how much a single array deviates from the remaining arrays within the experiment post-normalization (*dfArray*). We compare our findings to currently available metrics for FFPE samples using the DASL assay and show the benefit of removing arrays of questionable quality from an experiment where differential expression is the primary objective.

### Case study

The case study consisted of patients with resected HER-2 positive breast cancer who were enrolled in the adjuvant N9831 trial (NCT00005970), which was a Phase III trial where patients were randomized to three arms: (Arm A) doxorubicin and cyclophosphamide followed by weekly paclitaxel, (Arm B) same as Arm A but followed by 1 year of sequential trastuzumab, or (Arm C) same as Arm A but with 1 year concurrent trastuzumab started the same day as weekly paclitaxel [19]. Patient consent was obtained for additional translational work related to the tumor specimens and the institutional review board of all participating institutions approved the study. A total of 1632 samples from 1460 unique patients were labeled using the Whole-Genome DASL HT Assay and hybridized on the HumanHT-12 v4 Expression BeadChip. Patient samples were randomized onto 96-well plates, stratified by treatment arm, year on N9831 study and nodal status. The final dataset used herein consists of 1618 arrays after removing subjects that had withdrawn consent post data acquisition.

## Methods

### Model specification

We begin with a description of a basic statistical model for microarray data and will follow the notation as described by McCall and others [20-23]. The basic physical architecture of a microarray is that a specific probe is designed to bind to a specific RNA transcript. The RNA is extracted according to the manufacture’s protocol and hybridized to an array. The observed intensity *I*_*ij*_ of the i^th^ feature (i = 1, … , p) from the j^th^ sample (j = 1, …, n) is expressed

(1) $$I_{\mathrm{ij}}=K_{i}{}_{j}\times \theta_{\mathrm{ij}}\times \varphi_{\mathrm{ij}}+O_{\mathrm{ij}}$$

The term *O*_*ij*_ represents background intensity present in the data due to scanner inefficiencies and non-specific binding of probes. This background is typically subtracted from the data using vendor-specific methods or user specified packages. We leave it up to the user to specify which correction is to be used and simply move to the commonly used log-linear model form of (1)

(2) $$Y_{\mathrm{ij}}=\mu_{\mathrm{ij}}+S_{\mathrm{ij}}+\varepsilon_{\mathrm{ij}}$$

Where *Y*_*ij*_ denotes the intensity values after background correction, *μ*_*ij*_ = log_2_*θ*_*ij*_ represents the “true” relative amount of a feature hybridized to the array and is the primary parameter of interest in microarray experiments, *S*_*ij*_ = log_2_*K*_*ij*_ represents systematic biases, and *ε*_*ij*_ *=* log_2_*θ*_*ij*_ represents random variation with mean 0 and variance *σ*_*i*_ with the subscript indicating that the variance is feature specific.

The term *S*_*ij*_ represents an arbitrary bias function for the i^th^ feature on the j^th^ array and is assumed to be independent of the remaining parameters in equation (2). Examples of biases might be variations in sample dilution that would add a constant value to probes on the array, or other more complicated effects. The bias function is estimated using any number of user-specified normalization routines of which the most popular is quantile normalization and is used throughout this work [14]. We denote the post-normalized data as

(3) $$Y_{\mathrm{ij}}^{'}=Y_{i}{}_{j}-S_{\mathrm{ij}}=\mu_{\mathrm{ij}}+\varepsilon_{\mathrm{ij}}^{'}$$

### Review of other metrics

Bolstad et al. [24] and McCall et al. [20] introduced metrics to evaluate whether or not an array is an outlier based on the post-normalized data *Y*^′^_*ij*_. Bolstad’s first metric referred to as the *Relative Log Expression* (*RLE*) is defined as

$$\mathrm{RLE}=Y_{\mathrm{ij}}^{'}-\underset{j}{\mathrm{median}}\left(Y_{\mathrm{ij}}^{'}\right)$$

and compares a given array’s feature intensity relative to the median level of intensity for that feature across all j arrays. The array-specific distribution of *RLE* is used to determine if a particular array has predominately low- or high-expressed features as indicated by an overall shift. This metric is easily applicable to any microarray platform. However, for normalization routines that leverage probe-specific information such as loess, RLE ≅ 0 by definition so one does not expect to see large shifts. Moreover, the spread in the distribution of *RLE* is not independent of feature variance *σ*_*i*_^2^. This makes distribution summaries difficult to interpret for the purpose of outlier detection as an outlier for a particular feature can be masked by the other features with large variance.

The next metric, first introduced by Bolstad (*Normalized Unscaled Standard Error; NUSE*) and later modified by McCall (*Global NUSE; GNUSE*), was developed for evaluating the quality of Affymetrix array data. Both measure array quality relative to the standard error of the estimated feature abundance. In the case of Affymetrix, the primary feature of interest is gene level expression and is estimated by taking a robust average of probe sequences (average of 11 probes per gene on the HGU133plus2 array). Whereas *RLE* is used to look for overall shifts in the distribution of intensity between arrays, *NUSE* and *GNUSE* assess the variability of the estimated feature intensity across arrays and is defined as

$$\mathrm{GNUS}E_{\mathrm{ij}}=\frac{\mathrm{SE}\left(Y_{\mathrm{ij}}^{'}\right)}{\underset{j}{\mathrm{median}}\;\left(\mathrm{SE}\left(Y_{\mathrm{ij}}^{'}\right)\right)}$$

The two measures only differ in that the *GNUSE* metric uses distributional information on *Y*_*ij*_^'^ from a large collection of stored arrays to estimate the denominator *median*_*j*_(*SE*(*Y*_*ij*_^'^)) whereas *NUSE* re-estimates this for each new experiment. Regardless of which form is used, if the median *NUSE* or *GNUSE* for a particular array is high, this would be an indication that many of the features are behaving poorly and thus the array should be considered for removal. A value of 1.25 for the median *NUSE* or *GNUSE* has been suggested by McCall as a guideline for identifying bad arrays as this suggests that the variation for the array is 25% higher than an average array.

**Figure 2:**
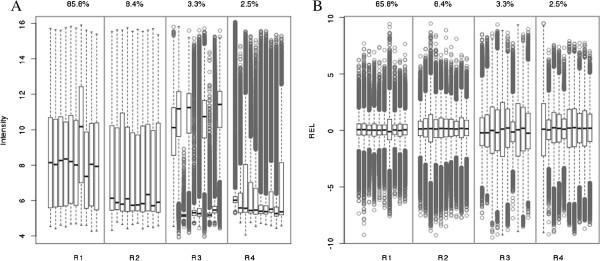
**Arrays from the breast cancer case study were grouped into four quadrants according to their IQR range (IQR = Q3-Q1) and skewness (skew = (Q2-Q1)/IQR; symmetric distributions will have skew=0.5).** Quadrant R1 consists of arrays with IQR>2 and skew>0.2. Quadrant R2 consists of arrays with IQR>2 and skew≤0.2. Quadrant R3 consists of arrays with IQR≤2 and skew>0.2. Quadrant R4 consists of arrays with IQR≤2 and skew≤0.2. The percent of the 1618 arrays that fall into each quadrant are summarized along the top of the figure. Panel (**A**) depicts pre-normalized intensity values; Panel (**B**) depicts the RLE metric.

One drawback for NUSE and GNUSE is that they are tailored towards the Affymetrix platform as multiple probes per gene are needed in order to estimate *SE (Y*^′^_*ij*_). For the Illumina platform, depending on the gene annotation used, between 45 to 55% of genes have only one probe per gene making calculation of *SE (Y*^′^_*ij*_) at the gene level meaningless for a high proportion of the array. Each probe on the Illumina array has on average 30 to 40 beads per probe that are used to quantify probe specific expression and various authors have leveraged this aspect of the design in their assessment of differential expression [25,26]. Potentially, one could also use the bead standard error as an estimate of *SE (Y*^′^_*ij*_) for the calculation of NUSE. There are two fundamental flaws to this approach. First, as shown in Figure 1, the NUSE metric is directly proportional to the median expression for an array. This indicates that arrays with samples having higher RNA concentrations will be penalized more than arrays with samples closer to the overall median. Secondly, the criteria for determining an array as poor quality is purely one-sided and does not detect arrays where RNA concentrations are at the lower limits of detection (Figure 1). This is especially important for FFPE samples as there is a wide level of expression patterns with many arrays towards the lower limit of detection (Figure 2).

Recently, Chow et al. described their quality assessment workflow using the *lumi* package developed by Du et al. for DASL arrays [18,27]. This workflow is summarized in Table 1. The main metric used in the *lumi* package is the “distance to the average array”, which we will define as

$$\mathrm{lumiOutlie}r_{j}=\mathrm{dissimilarity}\;\left(Z_{\mathrm{ij}}\text{,}\mathrm{Targe}t_{i}\right)$$

**Table 1:** Quality assessment strategies for Formalin Fixed Paraffin-Embedded tissues analyzed with Illumina’s DASL assay

**Mahoney et al.**	**Chow et al.**
Normalize Data	
Calculate *Stress* and *dfArray*	Calculate *Outlier* using un-normalized raw data
(Plot *Stress* vs *dfArray*)	
Stage 1: Remove arrays with *Stress* ≥ 1.5	Stage 1: Remove arrays with *Outlier* ≥ Th*median(*Outlier*) (Default *Th* = 2)
Renormalize data after removing bad arrays	Renormalize data after removing bad arrays
Calculate *dfArray* on renormalized data	Calculate *Outlier* on renormalized data
Stage 2: Investigate arrays with *dfArray* ≥ 2	Stage 2: Remove arrays with *Outlier* ≥ Th*median(*Outlier*) (Default *Th* = 2)
Final normalization after removing all outlying arrays	

Where *Z*_*ij*_ represents the feature mean centered and scaled pre- or post-normalized expression data for the i^th^ feature from the j^th^ array and *Target*_*i*_ represents a robust estimate of the feature mean across all arrays and is a correspondingly a pre- or post-normalized estimate (Table 1). The dissimilarity function used is either the Euclidean distance of the j^th^ array from the *Target* or one minus the correlation between the j^th^ array and the *Target*. The *lumi* package considers an array as an outlier whenever *lumiOutlier*_*j*_ > *Th*  ×  *median* (*lumiOutlier*_*j*_) , where *Th* is a user-specified threshold (default specified in the package is *Th* = 2). It is difficult to attribute a biologically meaningful interpretation of this metric in such a way as to make it easily transparent to the average researcher. Another drawback is that the threshold is defined relative to the current sample of arrays. Thresholds that are sample dependent are problematic in practice as they vary from batch-to-batch and provide no sense of global quality of an array beyond the average array within the current batch. If, for example, the average array is also of poor quality, the researcher is left with an experiment containing many poor arrays jeopardizing the validity of the study.

### Proposed methods

To address the shortcomings of the metrics purposed thus far, we propose two metrics that combine the essence of *RLE*, *NUSE/GNUSE*, and the *lum*i*Outlier*, yet are flexible enough to be implemented on a broad spectrum of microarray platforms with direct biological interpretation. Importantly for the analysis of archival tissues, the proposed metrics allow for the identification of poor arrays that have undue influence during the normalization process. Such arrays are fairly obvious to identify when evaluating data from fresh-frozen samples; however, it is less obvious to determine a threshold for determining poor samples with archival samples.

From Equation (3), *S*_*ij*_ can be viewed as an estimate for the amount that each feature on an array needs to be shifted for normalization; we refer to this as the *Stress* measure due to its similarity to the same concept in multidimensional scaling. The overall distribution of *Stress* captures the amount of deformation that was applied to an array during normalization. Since we are not concerned about arrays that differ from other arrays by a constant shift (e.g., the scanner was 50% brighter for one array compared to the others) the array-specific mean of *S*_*ij*_ is subtracted, which leads to our first metric called *Stress* and is defined as

$$\mathrm{lo}g_{2}\left(\mathrm{Stres}s_{j}\right)=\mathrm{median}\left(\left|S_{\mathrm{ij}}-\bar{S_{j}}\right|\right)$$

and is calculated across all i features on a specific array. The *log*_*2*_ is used here to indicate that the index will need to be transformed to the fold-change scale. Also, by taking the absolute value, features that are up or down regulated by “x-fold” are considered equally *Stress*ed. Various distributional summaries and figures can be generated on *Stress*_*j*_, but we found the median to be the most useful. Arrays can be rank ordered according to their *Stress* values, and the arrays with the highest or more disparate *Stress* values would be considered as suspect for inclusion in the study. As an example, if the median *Stress* of an array is 2, this would indicate that half of the features had to be adjusted by 100% or more relative to their initial values. For many studies, a 2-fold change is the biological effect size of interest. Any final result becomes highly suspect when it is of the same order of magnitude as the biases that were removed from the data.

Although *Stress* provides a biologically meaningful measure of how much the global distribution of a sample will change during normalization, it does not leverage feature-specific information. From Equation (3), and under the assumption that a preponderance of features on a microarray are not differentially expressed, an estimate of intensity for the i^th^ feature is simply the mean of that feature across the n arrays ${\hat{\mu }}_{i}=Y_{i}^{'}$. Let ${\hat{\mu }}_{i\left(-j\right)}$ denote the mean and $\mathrm{sd}\left({\hat{\mu }}_{i\left(-j\right)}\right)$ denote the sample standard deviation of the i^th^ feature by excluding the j^th^ array. We define our next metric *dfArray* (Deviation oF Array) as

(4) $$\mathrm{dfArra}y_{\mathrm{ij}}=\frac{Y_{\mathrm{ij}}^{'}-{\hat{\mu }}_{i\left(-j\right)}}{\mathrm{sd}\left({\hat{\mu }}_{i\left(-j\right)}\right)}$$

and is analogous to common diagnostic tests in linear regression modeling known as Cooks distance [28]. The numerator of *dfArray* is similar to the RLE metric proposed by Bolstad, but *dfArray* is scaled by the standard deviation of the remaining arrays so that features are scaled both within and across arrays as the distribution of each feature will have a mean of zero and a standard deviation of one. Recalculation of Equation (4) for each array is computationally tedious especially for large experiments and a more efficient approach is to replace ${\hat{\mu }}_{i\left(-j\right)}$ and $\mathrm{sd}\left({\hat{\mu }}_{i\left(-j\right)}\right)$ with their corresponding robust estimates, which only need to be calculated once. In this work, we used the median expression of the i^th^ feature in place of ${\hat{\mu }}_{i\left(-j\right)}$ and the median absolute deviation in place of $\mathrm{sd}\left({\hat{\mu }}_{i\left(-j\right)}\right)$. We have found various distribution summaries and figures of *dfArray* to be useful for quality assessment purposes, but as a single summary of this metric we use

(5) $$\mathrm{dfArray}=\underset{i}{\mathrm{quantile}}\left(\left|\mathrm{dfArra}y_{\mathrm{ij}}\right|,0.75\right)$$

as values that fall above or below ${\hat{\mu }}_{i\left(-j\right)}$ are viewed as equivalent errors. For this work we consider any array with 25% of the features having expression levels larger than twice the standard deviation above the median expression as suspect. This threshold can certainly be modified by the user and by expressing the cutoff in terms of standard deviations above the median expression level allows for a better reference of understanding amongst researchers with basic statistical training.

As we show in the results, *dfArray* is highly correlated with the dissimilarity metric used in the *lumi* package. Since the dissimilarity metric is used in clustering procedures, this indicates that arrays with a large *dfArray* index may be associated to clinical subclasses not accounted for in the normalization process. Our proposed quality assessment strategy for FFPE samples analyzed is outlined in Table 1 and the R package Stress.dfArray is freely available at http://mayoresearch.mayo.edu/mayo/research/biostat/splusfunctions.cfm.

## Results

### Distributional characteristics of arrays

As described above, the case study used throughout consists of 1618 HumanHT-12 v4 Expression BeadChip DASL assays that were generated as part of an ongoing breast cancer study that analyzed FFPE archival tissues. Boxplots of the *log*_*2*_ transformed intensity values showed that the quality of the data varied dramatically between the samples. Specifically, it was apparent that some of the samples failed completely, while there were other samples for which it appeared that some of the probes worked while other probes did not. Figure 2A displays box-plots of the pre-normalized expression values for 40 samples, representing various array qualities. For presentation purposes, samples were assigned to 4 array-quality groups based on the interquartile range (IQR = Q3 – Q1) and skewness (skew = (Q3-Q1)/IQR; symmetric distribution will have skew = 0.50) in order to represent the extremes in array quality and 10 representative samples are shown for each group. Approximately 15% of the 1618 FFPE samples examined exhibited large skewness (shown in quadrant R2), a small IQR (quadrant R3), or both (quadrant R4). Unlike data from fresh-frozen samples where only a couple of arrays might be poor and are obvious to detect, the distribution of intensity values from archival samples vary dramatically and there is not a clear threshold for determining which arrays are of poor quality.

### Association of quality metrics with array characteristics

**Figure 3:**
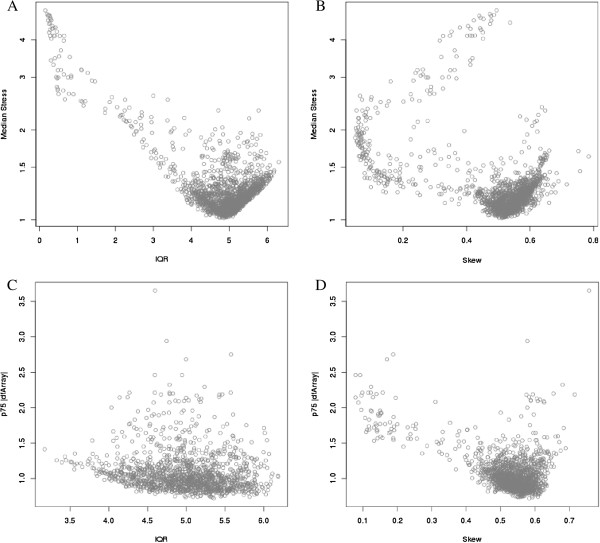
**Plot of IQR (A) and skewness (B) of un-normalized data versus Median *Stress *for all 1618 arrays.** Plot of IQR (**C**) and skewness (**D**) of un-normalized data versus median *dfArray* for the 1410 arrays with median *Stress* < 1.5.

The IQR and skewness thresholds used in Figure 2A to identify potentially poor-quality samples are ad hoc; however, they do provide a reasonable first-pass look into the data. As discussed above, *GNUSE* or *NUSE* cannot be applied as the majority of the features on the DASL array do not have multiple probes nor is there a comprehensive archive of the HumanHT-12 v4 Expression Beadchip DASL assay to define a reference distribution. Figure 2B displays box-plots of the *RLE* metric on the same set of arrays shown in Figure 2A. From our experiences, it is difficult to detect striking deviations in the RLE across good and poor-quality samples. Thus, we applied the metrics *Stress*, *dfArray*, and *lumi Outlier* in an attempt to identify poor-quality samples. First, we compared *Stress* against the IQR (Figure 3A) and skewness (Figure 3A); *Stress* was calculated using quantile normalization. Arrays with a low IQR have a high median *Stress* indicating that the global distribution for these arrays would require the most “stretching” during normalization (Figure 2A). Similarly, there is a general tendency for arrays with higher levels of skewness to also have high median *Stress* (Figure 3B). Using 1.5 as a threshold, we removed all arrays with median *Stress* ≥ 1.5 and subsequently calculated *dfArray* and further compared *dfArray* to IQR (Figure 3C) and skewness (Figure 3D). Even after removing arrays based on their global distributions using *Stress*, *dfArray* shows that arrays of questionable quality might remain. Specifically, we see that there are arrays with large IQR that might be of poor quality (Figure 3C). Additionally, there are arrays of various skewness levels that might be of poor quality (Figure 3D).

### Concordance of quality metrics

**Figure 4:**
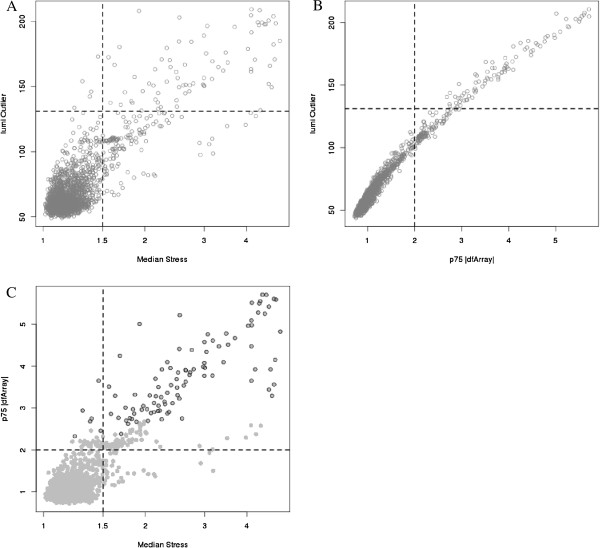
**Plot of *lumi Outlier *versus median *Stress *(A) and 75**^**th **^**percentile absolute value of *dfArray *(B) with reference dashed lines indicating the respective threshold for outlier detection for each method.** Plot of median *Stress* versus 75^th^ percentile absolute value of *dfArray* (**C**) with dashed lines indicating the respective threshold for outlier detection. Open circles indicate outliers as identified by *lumi Outlier*.

*lumiOutlier* has been proposed as a quality-control metric specifically for Illumina microarrays and thus we evaluated the concordance between *dfArray*, *Stress*, and *lumi Outlier*. As displayed in Figure 4A, *Stress* and *lumi Outlier* are correlated; the suggested threshold for outlier determination for each method is indicated. Using the suggested thresholds, *Stress* identified most of the samples that *lumi Outlier* did as well as additional arrays. Similarly, we see the direct association between *dfArray* and *lumi Outlier* as anticipated as both metrics are functions of Equation 4 (Figure 4B). Cleary, *dfArray* and *lumi Outlier* could be calibrated to the same scale, but *dfArray* is already on a scale of biological relevance as it expressed the deviation of an array in units of standard deviation of the features being measured. This is a natural metric for deciding intuitive threshold values a prior and holds across studies. Conversely, there is vagueness in determining a threshold value for a distance metric such as that used by *lumi Outlier* and the threshold is data dependent. Figure 4C displays the correlation between *dfArray* and *Stress* using quantile normalization on all 1618 arrays, where the open circles indicate arrays that are considered outliers by *lumi Outlier*. Arrays with the highest *Stress* also tend to have relatively large *dfArray* values and together they capture all the arrays that would be considered outliers by *lumi Outlier*.

### Benefit of conducting quality assessment on array data

**Figure 5:**
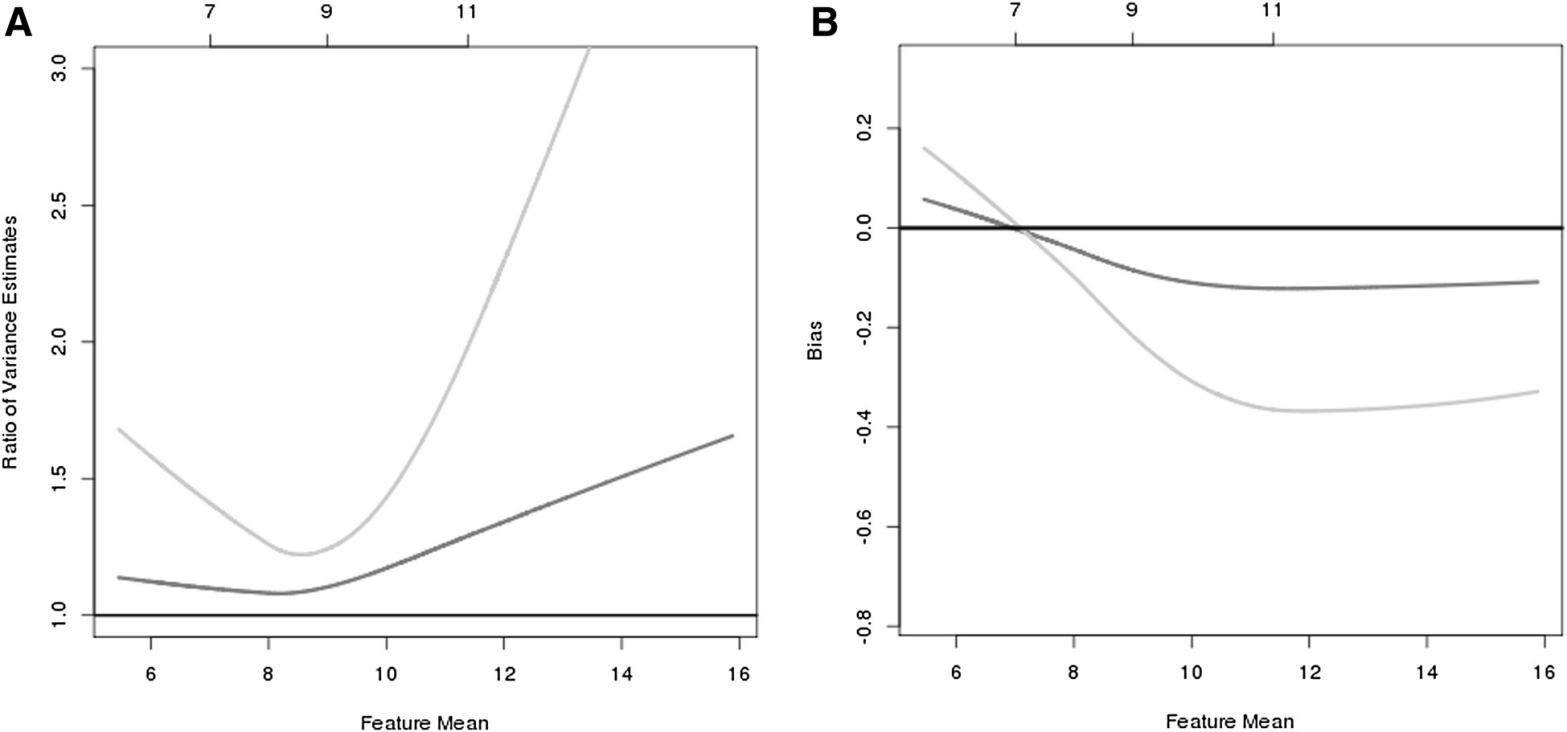
**The relative increase in feature variance (A) and bias in the estimated feature intensity (B) by *not*excluding any array ( light gray line ) and excluding *only*the arrays that were considered to be an outlier by the *lumi Outlier *( medium gray line ).** The 25^th^, 50^th^, and 75^th^ percentiles of feature intensity for the 1378 arrays are listed along the top of the figure and the reference lines at 1 (**A**) and 0 (**B**) represent the reference samples.

Lastly, to understand the impact of questionable arrays, we estimated the bias and variance of feature level expression by considering the 1378 arrays that were of good quality for all three metrics (*Stress*/*dfArray*/*lumiOutlier*) as the reference sample. There were 100 arrays that were considered as outliers by all three methods and 140 arrays that were considered as outliers only by *dfArray* and *Stress*, but not *lumi Outlier*. At each stage, when additional samples are added to the reference sample of 1378 arrays, the data were re-normalized using quantile normalization and the corresponding feature mean and variance was recalculated and compared to the estimates from the reference sample. Figure 5A displays the ratio of the feature variances relative to the feature variance of the 1378 reference arrays when additional arrays are included. When none of the questionable arrays are excluded (i.e., adding the 100 + 140 arrays that were considered as bad quality for any of the three methods; light gray line), the feature variance is at least 25% larger than the variance in the reference sample and becomes increasingly larger for both low- and high-intensity values. When excluding only the 100 arrays that were considered as outliers by all three methods (or when including the 140 arrays that were considered questionable by only *Stress*/*dfArray*) , Figure 5A shows that the relative increase in variance is approximately 5% to 10% higher for low-intensity features but is 50% larger for high-intensity features (dark grey line). Similarly, the bias in the estimated feature abundance is highest when including all of the questionable arrays and is lower, but still present, when excluding only the 100 arrays that were considered questionable by all three methods. Again, biologically we anticipate that only a relatively small number of features are truly differentially expressed between samples and therefore we would not expect to see any shifts in the estimated mean or variance of feature intensities when including or excluding any number of arrays.

## Discussion

The use of microarrays in understanding disease pathogenesis has seen extraordinary growth over the last decade. Historically, data generated by this technology has been used for class comparisons (comparing gene expression profiles between known disease states), class prediction (prediction of disease state), and class discovery (identification of new subclasses of disease base on gene expression profiles). Recently, interest has moved from the bench to the bed side where treatment decisions based on gene-expression profiles obtained from microarrays are being considered [29]. In fact, this is the objective of the current case study; to define a molecular signature to predict response to trastuzumab for HER2-positive breast cancer patients.

As the use of microarrays has increased, so to have the concerns about the validity of this technology [30-33]. Some of these concerns broadly revolve around proper analytical methods, the concordance of results between publications, centers, or laboratories, and the concordance of results between different platforms, to name just a few. Several research initiatives have formed over the years to investigate these concerns dating back to the early days of “Affycomp” [34] to the more recent formation of the External RNA Control Consortium and the MicroArray Quality and Countrol projects [30]. These efforts have facilitated greater communication between researchers as well as the development of standard practices to increase the validity of microarray technologies. The overarching theme resulting from these efforts is that microarray technologies are reliably reproducible across many different settings with proper laboratory procedures, data handling, and scrutiny. Several investigations have reported on the gain in analytic efficiency when poor-quality microarrays are removed [20,35]. However, most and if not all of this work has centered on analysis of fresh-frozen samples.

Analysis of archival tissues presents a new challenge and is complicated by poor RNA quality and significant variation among FFPE samples that have been preserved over the course of many years and under different conditions. As we have shown here, this variation in sample quality for FFPE samples creates large variation in the expression profiles across arrays that are typically not seen when dealing with fresh-frozen samples. This has spurred many questions regarding the normalization, quality assessment, and analysis of array based studies using FFPE samples [10].

The choice of normalization routine may have an impact on downstream analyses when it comes to FFPE samples. Many of the FFPE samples in the present study exhibit a high prevalence of “dead probes” where little or no signal is generated beyond background. Many of the more popular normalization routines (e.g., quantile, loess) used in practice were developed on data where the prevalence of dead probes was very small. Therefore, we believe additional studies are required to determine the best normalization strategy for data that is generated from the FFPE samples.

It is important to note that normalization is not the end all step to preprocessing microarray data and certainly not a solution for poorly-designed studies. Assessing the quality of microarray data is essential and the two metrics proposed here, *Stress* and *dfArray*, are easily applicable to any microarray platform for this purpose. For studies using FFPE samples, removing arrays that are of poor quality from the normalization process reduces the bias in the estimated feature abundance and the noise level in the data and thus increases the ability to detect biologically-meaningful differences. Some have suggested that the information provided by the quality metrics could also be used to weight downstream analyses towards arrays with better quality [36]. This is potentially a viable option for studies using FFPE samples, but more research is needed. We anticipate that the arrays identified by the *Stress* metric as being an outlier have the greatest influence on the normalization process and therefore will need to be excluded. However, the *Stress* metric could be recomputed after removing outliers and either the newly-computed *Stress* metric or *dfArray* could be used to down weight arrays during differential-expression analyses.

As more high dimensional data become publicly available, there is an increasing interest to pool data across studies, or at the very least, mine these repositories for promising biomarker signatures prior to initiating a research project. At our institution, such an endeavor is being implemented through the creation of the Biologically Oriented Repository Architecture (BORA), which is an informatics warehouse of “-omics” data that is linked to the tissue pathology and clinical characteristics of the patient. These types of initiatives require robust quality metrics to accurately assess high dimensional data across multiple studies especially when the data has been preprocessed and summarized prior to storage.

## Findings

Two robust quality control metrics are presented that provide the end-users with valuable information regarding the quality of the arrays within their study. These metrics are directly applicable to any high-dimensional platform and can be easily implemented into preprocessing pipelines.

## Availability and requirements

Package name: Stress.dfArray

Package source: http://mayoresearch.mayo.edu/mayo/research/biostat/splusfunctions.cfm

Requirements: R-2.14.0 or later (http://www.r-project.org/)

## Abbreviations

RLE: Relative Log Expression; NUSE: Normalized Unscaled Standard Error; GNUSE: Global Normalized Unscaled Standard Error; dfArray: Deviation of array; DASL: cDNA-mediated Annealing Selection extension and Ligation; Th: Threshold; FFPE: Formalin-Fixed, Parriffin-Embedded; Q1,Q3: First and third quartiles; IQR: Interquartile range; SE: Standard Error; sd: Standard deviation.

## Competing interests

The authors declare that they have no competing interests.

## Authors’ contributions

DWM, TMT, SKA and JE-P developed the statistical methodology. JJ, JPAK, MMR and EAP provided guidance on the statistical methodologies. MMR and EAP provided the case study. All authors read and approved the final manuscript.
